# The Role of Oxidative Stress in NAFLD–NASH–HCC Transition—Focus on NADPH Oxidases

**DOI:** 10.3390/biomedicines9060687

**Published:** 2021-06-17

**Authors:** Daniela Gabbia, Luana Cannella, Sara De Martin

**Affiliations:** Department of Pharmaceutical and Pharmacological Sciences, University of Padova, 35131 Padova, Italy; daniela.gabbia@unipd.it (D.G.); luana.cannella@studenti.unipd.it (L.C.)

**Keywords:** NASH, NAFLD, hepatocellular carcinoma HCC, NADPH oxidases, NOX, oxidative stress

## Abstract

A peculiar role for oxidative stress in non-alcoholic fatty liver disease (NAFLD) and its transition to the inflammatory complication non-alcoholic steatohepatitis (NASH), as well as in its threatening evolution to hepatocellular carcinoma (HCC), is supported by numerous experimental and clinical studies. NADPH oxidases (NOXs) are enzymes producing reactive oxygen species (ROS), whose abundance in liver cells is closely related to inflammation and immune responses. Here, we reviewed recent findings regarding this topic, focusing on the role of NOXs in the different stages of fatty liver disease and describing the current knowledge about their mechanisms of action. We conclude that, although there is a consensus that NOX-produced ROS are toxic in non-neoplastic conditions due to their role in the inflammatory vicious cycle sustaining the transition of NAFLD to NASH, their effect is controversial in the neoplastic transition towards HCC. In this regard, there are indications of a differential effect of NOX isoforms, since NOX1 and NOX2 play a detrimental role, whereas increased NOX4 expression appears to be correlated with better HCC prognosis in some studies. Further studies are needed to fully unravel the mechanisms of action of NOXs and their relationships with the signaling pathways modulating steatosis and liver cancer development.

## 1. Introduction

Non-alcoholic fatty liver disease (NAFLD) includes a wide range of hepatic disorders characterized by a progressive accumulation of lipids in the hepatocytes. This disease comprises different conditions, ranging from uncomplicated steatosis to its severe complication non-alcoholic steatohepatitis (NASH) [1], a chronic liver disease which, besides the presence of hepatocellular lipids, presents inflammation and injury of the hepatic parenchyma. NAFLD is a quite common condition, since it affects one-fourth of the world population [2], and it has been estimated that the progression from NAFLD to NASH occurs in nearly one-third of NAFLD patients [3], although some studies reported that this transition is much more frequent [1]. It is well known that the progression from NAFLD to NASH is influenced by several factors, including health conditions, such as diabetes and obesity, as well as genetic and environmental factors [3]. Numerous studies have tempted to describe in detail the mechanism(s) underlying the transition from NAFLD to NASH. Among the different theories which have been postulated, there is a consensus that lipotoxicity, mitochondrial dysfunction, and oxidative stress play a pivotal role in this process [4]. 

It has been extensively demonstrated that NASH is a fundamental factor in the etiology of liver fibrosis, cirrhosis, and hepatocellular carcinoma (HCC), the latter being the most common form of liver cancer [1,2,3]. HCC, which can develop in NASH patients either because of or in the absence of cirrhosis [5], remains a global health challenge, due to its growing incidence and limited therapeutic options. Worldwide, it represents the third leading cause of cancer-related deaths, and it has been estimated that more than 1 million cases per year will be diagnosed by 2025 [6,7]. Despite the recent advances in cancer therapy, HCC patients still have a low overall survival, and limited therapeutic options, such as surgical resection, liver transplantation, and a restricted number of kinase or immune checkpoint inhibitors, are currently approved, although several innovative biological drugs are in the pipeline [8]. The most important risk factors for NASH–HCC transition are the severity of liver fibrosis, male gender and age, and metabolic factors, such as obesity, diabetes, insulin resistance, and metabolic syndrome. Nonetheless, also slim NASH patients could develop liver cirrhosis and cancer, even in the absence of metabolic dysfunctions. Numerous studies described the specific mechanisms of the NASH–HCC transition, unraveling that metabolic and oxidative stress, immune and endocrine alterations, and pathological inflammatory responses are fundamental players in this process [6]. In light of these considerations, this review aims at summarizing the current knowledge about oxidative stress involvement in the NAFLD–NASH–HCC transition, focusing on the role of the enzymes NADPH oxidases (NOXs). 

## 2. The Role of Oxidative Stress in NAFLD and NASH

As hinted in the Introduction, the role of oxidative stress in liver diseases associated with metabolic imbalances, such as NAFLD and NASH, is well established, and numerous studies with precise mechanistic purposes have been recently published (see [9] for a recent review). However, the complex interplay between oxidative stress mediators and the mechanisms causing and sustaining liver dysfunction is still far to be completely understood. In hepatic steatosis, oxidative stress has been strictly linked to immune cell responses [10], since both preclinical and clinical evidence demonstrated that NASH is characterized by the infiltration of adaptive immune cells in the liver and by the presence of circulating antibodies directed toward antigens taking origin from oxidative stress. Indeed, lipid peroxidation, which is a common feature of NAFLD and NASH [11], generates oxidized phospholipids, such as phosphocholine on oxidized phospholipids and oxidized cardiolipin, and reactive aldehydes, among which the most studied and best characterized are malondialdehyde (MDA) and 4-hydroxynonenal (4-HNE). Interestingly, both of these oxidative stress-derived molecules act as direct inducers of hepatic inflammation [12] and as antigenic adducts named oxidative stress-derived epitopes or oxidation-specific epitopes (OSEs), by reacting with self-cellular macromolecules [13]. Indeed, oxidative stress molecules released from damaged parenchymal and non-parenchymal liver cells act as damage-associated molecular patterns (DAMPs) by interacting with membrane receptors, such as the Toll-like (TLRs) and other receptors expressed on immune system cells, with the final aim of alerting the host and promote their removal [14], thereby activating innate immunity responses [15]. This mechanism is linked to the generation of macromolecular adducts by lipid-derived reactive carbonyl species (RCS), a wide variety of break-down products characterized by the presence of a carbonyl moiety and high chemical reactivity [16]. Therefore, the peroxidation of lipids, besides altering their physiological functions, promotes the formation of these macromolecular neo-epitopes, named OSEs, whose presence has been observed in lipoproteins, dying cells, and extracellular vesicles. Increased presence of OSEs generated by MDA, 4-HNE, and oxidized phospholipids has been extensively demonstrated in the diseased liver (see [13] for an exhaustive review), and a correlation between their abundance and the severity of NAFLD and NASH has been demonstrated by both experimental [12] and clinical studies [17]. Coherently, targeting oxidized phospholipids has been suggested as a therapeutic strategy for the management of hepatic inflammation [18] and, more recently, for NASH [19]. Recently, it has also been demonstrated that OSEs can activate the innate immunity receptor for advanced glycation end products (RAGE) [20], a receptor interacting with ligands highly present during inflammation. Interestingly, two different pathways are activated upon interaction with RAGE, i.e., NADPH oxidases, resulting in the further production of reactive oxygen species (ROS), and the nuclear factor (NF)-κB pathway, which leads to a sustained pro-inflammatory and pro-fibrotic response [21]. Therefore, the engagement of RAGE exerted by its ligands helps the sustainment of inflammation, finally leading to tissue injury. However, despite the prominent role of RAGE in other forms of liver injuries [22], its importance in NAFLD and NASH remains controversial, since conflicting evidence has been obtained in animal [23] and clinical studies [24].

Finally, besides their role in the innate immune response, it must be reminded that OSEs acts as neoantigens and play a pivotal role in the activation of adaptive immune responses, as recently reviewed [10]. The starting point of the definition of this OSE role, leading to the mechanistic studies performed later, was the results of three independent studies, in which elevated titers of IgGs directed towards OSEs were detected in nearly a half of adult patients with NAFLD/NASH [25,26] and an even higher percentage (60%) of children affected by NASH [27], indicating that also adaptive immune reactions are involved in the oxidative stress–immune system interplay. Furthermore, a particular subset of adaptive immune cells, i.e., regulatory T cells (Tregs), demonstrated to be modulated by oxidative stress in NAFLD/NASH. Tregs, which are a subset of T cells essential for maintaining peripheral tolerance, preventing autoimmunity, and limiting chronic inflammatory diseases [28], have proven to be more prone to mechanisms of programmed cell death, such as apoptosis, in a fatty liver with respect to a normal healthy liver [29]. Furthermore, a recent study underlined the importance of Tregs in NAFLD and NASH, providing clinical evidence of their role in liver damage progression [30]. 

**Figure 1:**
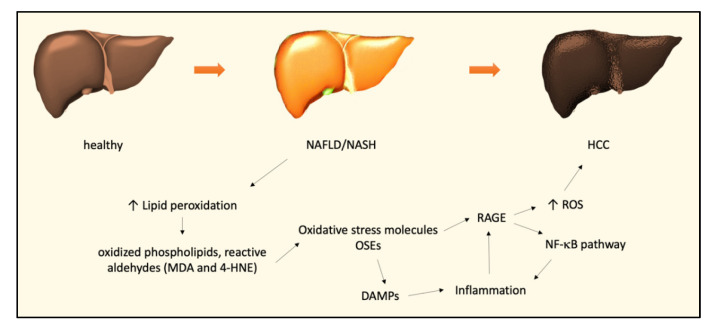
Molecular oxidative stress-related mechanisms involved in the NAFLD–NASH–HCC transition. Abbreviations: non-alcoholic fatty liver disease, NAFLD; non-alcoholic steatohepatitis, NASH; hepatocellular carcinoma, HCC; malondialdehyde, MDA; 4-hydroxynonenal, 4-HNE; oxidation-specific epitopes, OSEs; receptor for advanced glycation end products, RAGE; reactive oxygen species, ROS; damage-associated molecular patterns, DAMPs.

Taken together, these observations are consistent in picturing a fascinating scenario, in which oxidative stress and innate and adaptive immunity cooperate in generating and supporting hepatic inflammation, favoring the transition from NAFLD to NASH (Figure 1).

## 3. The Role of Oxidative Stress in the NASH–HCC Transition

Epidemiological data clearly indicate that NAFLD/NASH causes a dramatic increase in the prevalence of HCC development [31], being NASH the etiological cause of HCC, which develops most rapidly among patients who are in a list for liver transplantation in the USA [32]. As stated in the Introduction, it is worth reminding that limited therapeutic options for HCC are available, characterized by low efficacy, although a large effort has been made to explore novel targeted therapies, and numerous experimental and clinical studies are currently ongoing [33]. However, a detailed characterization of the mechanism by which NASH initiates and sustains the neoplastic transition of hepatic cells is warranted to identify novel therapeutic targets and innovative strategies to prevent HCC in NASH patients. 

As for NAFLD and NASH, numerous studies have tried to unravel the role of oxidative stress in HCC development. The increase of oxidative stress in liver parenchymal cells has been observed in and linked to HCC, but both the detailed mechanisms and the overall impact of this specific issue remain to be fully elucidated [34]. It is well known that ROS, such as hydrogen peroxide, can cause either point mutations or larger lesions in the genome [35]. A recent study investigated in detail the role of oxidative stress-related enzymes and receptors in hepatic carcinogenesis, identifying three factors (thioredoxin reductase-1, glutathione reductase, and the transcription factor Nrf2) as major players in HCC development [36]. These authors concluded that this process is the results of a complex interplay between different factors. Indeed, although it is well known that the antioxidant systems constitute an integrated, finely tuned network able to effectively prevent carcinogenesis by protecting healthy cells, the role of oxidative stress is controversial in existing cancers, where ROS are definitely part of the tumor microenvironment (TME) [37], and the antioxidant network likely plays both anti- and pro-cancer roles. In this context, ROS are active players in cancer development, exerting apparently contradictory effects, i.e., either the stimulation of tumorigenesis and cancer cell proliferation or the induction of cell death. An indirect confirmation of the anticancer role of oxidative stress is the adaptation of tumor cells to antioxidant insults, for example by increasing NADPH via the pentose phosphate pathway (PPP) [38], this being also an emergent mechanism of drug resistance exploited by cancer cells [39]. In this context, it is clear that dissecting the role of NADPH oxidases, which are both NADPH consumers and ROS generators, is of outstanding interest.

## 4. NOXs and the NASH–HCC Transition

The NOX family comprises membrane-bound enzymatic complexes representing cytoplasmatic generators of ROS. At variance with other ROS-generating entities, such as mitochondria, for which ROS are essentially byproducts, NOXs have the precise role of directly catalyzing O_2_^−^ production from O_2_ by transferring electrons from NADPH across biological membranes. Therefore, these enzymes can regulate many redox-sensitive signaling pathways [40]. Since oxidative stress and ROS increase have been related to NASH and HCC promotion, many studies have focused on unraveling the role of NOXs in these diseases. 

**Figure 2:**
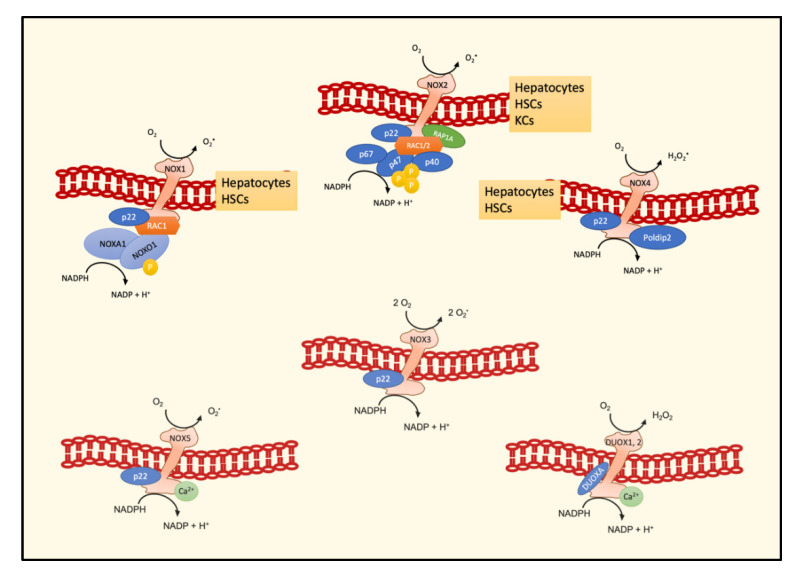
Schematic representation of subunits forming different NOX active enzymes and their main localization in hepatic cell types. Abbreviations: NADPH oxidase, NOX; hepatic stellate cells, HSCs; Kupffer cells, KCs.

In humans, the NOX family comprises seven members, namely, NOX1–5, DUOX1, and DUOX2, variable for tissue expression levels and for mechanisms of activation. In turn, each isoform comprises several subunits, different in number and peculiar activity (Figure 2). The isoforms of main interest in the liver are NOX1, NOX2, and NOX4. The expression of these three isoforms has been demonstrated in both hepatocytes and HSCs, whereas Kupffer cells mainly express NOX2 [41]. 

### 4.1. NOX1

NOX1 is formed by a RAC1 subunit, a membrane regulatory subunit called p22phox, which is also present in other isoforms, and an activator subunit (NOXA1) and is constitutively active, because its cytosolic subunit NOXO1 does not require phosphorylation to activate the complex. NOX2, the first isoform which has been described, is characterized by a RAC subunit, three cytosolic (p47phox, p67phox, p40phox) and one membrane (p22phox) regulatory subunits, and a catalytic subunit (gp91phox). The assembly of the three cytosolic subunits p47phox, p67phox, p40phox is required for the enzymatic activity, and the common subunits are interchangeable for these two isoforms. On the contrary, NOX4 consists only of two subunits, i.e., p22phox and Polidip2, which have to be bound to generate a fully functional enzyme [42]. Notably, by means of the CRISPR/Cas9 technique, it has been recently demonstrated that the knockout of p22phox leads to the of loss of NOX1 and NOX4 activity, but not of NOX5 activity [43].

Several NOX isoforms are known to play an important role in sustaining the progression of various chronic liver diseases, e.g., NAFLD, NASH, and liver fibrosis [42,44], by increasing oxidative stress through the production of ROS, which are a main feature of liver damage [45]. Interestingly, it has been demonstrated that Western diet consumption and metabolic syndrome can upregulate the expression of some NOX isoforms, thereby inducing hepatic inflammation and oxidative stress. In particular, it has been observed that a diet rich in fructose can upregulate the expression of both NOX2 and NOX4 in the liver and adipose tissue, whereas a high-fat diet, besides causing the hepatic accumulation of lipids, upregulates NOX2, p47phox, and NOX4 in the adipose tissue and NOX4 in the liver [46,47]. Furthermore, to strengthen the observed correlation between NOX and hepatic liver accumulation, a study investigated the existence of single-nucleotide polymorphisms (SNPs) in the genes encoding NOX4 and p22phox (*CYBA*) and observed an association between the rs3017887 SNP of *NOX4* and a higher ALT concentration in NAFLD patients and also between the AA genotype in the CYBA-675 T/A *CYBA* polymorphism and higher triglyceride and lower HDL levels in NASH patients [48]. NOX activity has been linked to the activation of the aryl hydrocarbon receptor (AHR) [49], a nuclear receptor controlling the expression of the enzyme CYP1A1 [50]. This observation is of note, since it has also been demonstrated that this enzyme protects mice from Western diet-induced NAFLD [51]. 

**Figure 3:**
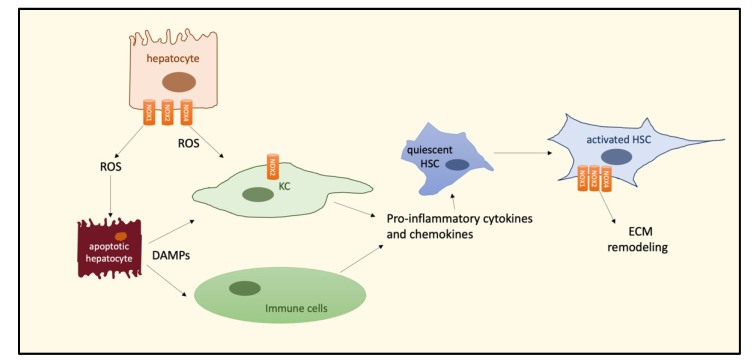
Schematic representation of NOX-induced ROS pathways leading to ECM remodeling during HCC development. NOXs isoforms are differently expressed in hepatic cells involved in NASH–fibrosis–HCC progression. Abbreviations: NADPH oxidase, NOX; hepatic stellate cell, HSC; Kupffer cell, KC; reactive oxygen species, ROS; damage-associated molecular patterns, DAMPs; extracellular matrix, ECM.

Liver fibrosis is also known as a trigger of oxidative stress and NOX upregulation. Indeed, an increased hepatic expression of NOX1, NOX2, and NOX4 has been demonstrated in mouse models of liver fibrosis [44], which was positively correlated with an exaggerated ROS production. NOX2, expressed in KCs and neutrophils, is responsible for their phagocytic activity and inflammation, while other nonphagocytic NOXs, e.g., NOX4, sustain oxidative injury and wound healing. As outlined before, chronic liver injury in NASH leads to liver fibrosis by different and complex mechanisms, among which the activation of HSC and HSC transition to the myofibroblast phenotype, responsible for increased production of extracellular matrix (ECM) proteins, play a pivotal role. Accumulating evidence suggests that NOXs are involved in HSC activation and apoptosis of hepatocytes, essential steps for initiating the fibrogenesis process [52]. Moreover, ROS-induced apoptosis of hepatocytes induces the release of damage-associated molecular patterns (DAMPs), able to activate KCs and recruit immune cells, thus increasing the production of cytokines and chemokines leading to HSC activation (Figure 3).

Several studies have suggested a role of NOX-derived ROS in tumorigenesis, and a detailed description of the signaling pathways by which they are involved in the neoplastic transition, such as protein kinase C, JAK–STAT, MAPK, and AKT pathways, has been performed. Furthermore, persistent liver inflammation has been linked to poor prognosis and aggressiveness of HCC [53,54]. Interestingly, it has been suggested that NOX-derived ROS could mediate TGF-β effect in tumorigenesis. The role of TGF-β in tumorigenesis is controversial, since it induces both NOX1 and NOX4 expression in hepatocytes, acting as a tumor suppressor in the early phases of tumor development but also promoting invasiveness and metastasis in advanced cancer. TGF-β-mediated NOX4 effect are involved in senescence and apoptosis of mutated hepatocytes through the regulation of STAT5 pathway and the pro-apoptotic genes *PUMA*, *BIM*, and *BMF*. At variance, NOX1 could activate the EGFR pathway, conferring partial resistance to TGF-β-mediated NOX4 upregulation and apoptotic effects, by modulating PI3K/AKT and ERKs pathways [53]. These observations demonstrate that the NOX1 and NOX4 isoforms could play opposite roles in the neoplastic process and likely regulate hepatic cancer growth in opposite ways. Therefore, it is reasonable to hypothesize that the balance of their activities could decide the fate of cancer cells, which could escape the TGF-β-mediated suppressor effect by switching this balance. Accordingly, a recent study has analyzed the expression of NOX isoforms in the liver of 377 HCC patients and 21 healthy donors to investigate whether some specific isoform could help to predict patients’ prognosis. Increased hepatic expression of NOX1, NOX4, NOX5, DUOX1, and DUOX2 was observed in HCC patients with respect to healthy donors. Interestingly, higher mRNA levels of *NOX4* and *DUOX1* correlated with a prolonged overall survival, whereas higher levels of *NOX1*, *NOX2* and *NOX5* were associated with a poor overall survival [54,55]. These findings further support other previous evidence of a different role of each NOX isoforms in HCC development [56,57,58]. However, further studies are needed to completely understand the role of the different NOX isoforms in liver cancer, keeping in mind that, as suggested, their functions might change in the different phases of tumor progression. 

In the liver NOX1, the non-phagocytic homolog of NOX2, can promote HSC proliferation through the PI3K/Akt pathway, thus prompting fibrosis development. This was demonstrated in an experimental model of fibrosis obtained by bile duct ligation [59]. Moreover, upregulation of NOX1 was demonstrated by Matsumoto and collaborators both in mice fed a high-fat diet (HFD) and in NASH patients. This was due to a Toll-like receptor 4 (TLR4)-dependent activation, consequent to an increase in palmitic acid. They also observed that NOX1-deficient mice displayed an attenuation of NAFLD-induced liver damage [59]. Furthermore, a recent study unraveled that the protective effect of HDL cholesterol in the liver is accompanied by a reduction of NOX1 expression and, consequently, oxidative stress [60]. Taken together, these results are consistent in indicating a detrimental effect of NOX1 expression and activation in NAFLD and its transition toward NASH.

As far as NOX1 role in liver cancer is concerned, a recent study investigating different NOX isoforms in a liver cancer mouse model demonstrated that NOX1 mediates DEN-induced hepatocarcinogenesis, since its ablation in macrophages inhibited cancer growth by affecting their ability to produce inflammatory cytokines. Notably, NOX4 ablation has no effect on DEN-induced carcinogenesis. Furthermore, ROS produced in macrophages mediated by NOX1 sustain survival and growth of oncogene-harboring mutant hepatocytes through STAT3 and ERK signaling pathways, thus promoting HCC development [61]. Accordingly, an increased level of NOX1 has been correlated with poor prognosis of HCC patients [54,62]. 

Interestingly, it has been observed that the combination of an immune checkpoint inhibitor (anti-PD1) with the NOX1 inhibitor GKT771 (Genkyotex) displayed an additional antitumor effect in an experimental model of colon carcinoma [63,64], thereby opening new perspectives for the use of NOX1 inhibitors as an adjuvant therapeutic option in solid tumors, including HCC. Indeed, the antitumor effect of GKT771 has been confirmed in a mouse model of HCC induced by DEN, where also a reduction of angiogenic markers and proinflammatory cytokines was observed. Therefore, the anticancer effect was attributed to an effect on macrophages polarization and to attenuation of fibrosis, crucial events in HCC development and progression [65]. A recent study found that NOX1, at variance with other NOX isoforms, is regulated by serine hydroxymethyltransferase 1 (SHMT1). This study showed that NOX1 acts both as the downstream target of SHMT1 and as a mediator of SHMT1 effects in HCC cells. These findings are of particular interest, since SHMT1 critical role as a tumor suppressor has been recently demonstrated in many human cancers and is exploited by regulating some factors related to epithelial–mesenchymal transition (EMT), e.g., vimentin and E-cadherin, which are strongly involved also in HCC development. Notably, SHMT1 resulted to be downregulated in NASH [66,67]. 

### 4.2. NOX2

The role of NOX2 in liver diseases such as NAFLD/NASH has long been studied, leading to interesting results. The study of Matsumoto and collaborators cited above reported a slight increase in the phagocytic NOX2 isoform in NASH mice, with a preferential expression in liver sinusoidal endothelial cells (LSECs) [59]. Notably, NOX2 seems to play a pivotal role in HFD-induced steatosis and insulin resistance [68]. This process could involve the activation of TLRs induced by the increased palmitate levels in the gut, which are in turn caused by NAFLD-related dysbiosis. NOX2 activity increases TGF-β phosphorylation due to increased peroxynitrite levels, thereby promoting liver fibrosis [69]. Interestingly, NOX2 has been linked to LSECs premature senescence, a condition associated with fibrogenesis [70]. In CCl_4_-induced liver fibrosis, the reduction of oxidative stress obtained by the knockdown of *NOX2* inhibited premature senescence, thus attenuating fibrogenesis. Another study demonstrated that NOX2 activation induced by an environmental toxin leads to miR21 upregulation in KCs, which in turn promotes the release of proinflammatory cytokines, HSC activation, and fibrosis. This study opens new perspectives on the “two-hit theory” formulated to explain NAFLD transition to NASH, pointing a light on the possible role of environmental contaminants acting as a “second hit”, thereby exacerbating liver damage by a NOX-2-dependent pathway [71]. 

A recent study demonstrated that HCC cells could induce macrophage polarization to the M2 tolerant phenotype, thus prompting cancer proliferation. This transition is mediated by a NOX2-dependent ROS increase that is in turn modulated by the high-mobility group box 1 (HMGB1)/TLR2/autophagy axis [72]. It should be noticed that HMGB1 is a prototypical ligand of RAGE, whose importance in NAFLD–NASH–HCC transition has been underlined before [21]. Another study analyzing hepatic tumor cells and coupled non-tumor hepatocytes in 134 HCC patients observed increased NOX2 cytoplasmatic levels in tumor cells with respect to their normal counterparts [55]. Its expression seems to affect cancer progression and metastasis, being implicated in the modulation of KRAS–RAF oncogenic signaling [54]. Yoshida et al. observed that the NOX subunit p22phox is expressed in preneoplastic hepatic foci during HFD-induced hepatocarcinogenesis, and the mineralocorticoid spironolactone in combination with β-glycosyl isoquercitrin is able to prevent these steatosis-induced precancerous lesions by reducing p67phox expression and the number of p22phox-positive cells [73]. 

Taken together, these findings depict a complex picture, indicating a peculiar role of NOX2-generated ROS in sustaining NAFLD transition to NASH. Notably, the consequent activation of inflammatory pathways typically occurring in NASH could help the transition of hepatic macrophages to pro-tumoral tolerant phenotypes, thereby sustaining HCC development, also by the direct activation of peculiar oncogenic pathways.

### 4.3. NOX4

In the liver, the NOX4 isoform is predominantly expressed in hepatocytes. Among the three NOX isoforms described in this review, NOX4 is undoubtedly the one offering the most puzzling results regarding its role in the fatty liver and the NAFLD–NASH–HCC transition. A study performed by Battaieb and collaborators demonstrated that its cell-specific deletion is able to reduce HFD-induced hepatic injury and fibrosis [74], even though another study failed to observe any NOX4 alteration in a similar mouse model [59]. In addition, the role of NOX4 in NASH–HCC transition remains controversial, since opposite results regarding the expression and activity of NOX4 in HCC development and patients’ survival have been reported in different studies. Indeed, it has been demonstrated that TGF-β displays its proapoptotic activity in hepatocytes by inducing NOX4 transcription, since NOX4 impaired activity caused resistance of HCC cells toward apoptosis [75]. Consistently, Yoshida et al. observed that supplementation with the NOX inhibitor APO downregulated the *p22pho*x gene in the hepatocytes of precancerous foci, reduced cell proliferation, and increased apoptosis [42]. Recently, by a dual in vitro/in vivo preclinical approach, it has been demonstrated that NOX4 deletion accelerated liver regeneration in mice [76], thereby underlying the pivotal role of this enzyme in hepatic cell proliferation. On the contrary, different results were obtained when considering the relationship between HCC and hypoxia. As other solid tumors, HCC is characterized by a strongly hypoxic microenvironment, due to increased oxygen consumption, that prompts tumor invasiveness, angiogenesis, and metastasis through the activation of hypoxia-inducible transcription factor-1 (HIF-1) that favors cancerous adaption to hypoxia [77]. Hypoxia induces NOX4 expression, and *NOX4* deletion is able to counteract hypoxia-induced GLI1-dependent epithelial–mesenchymal transition (EMT) and invasion of HCC cells [78]. Moreover, the NOX1/4 inhibitor setanaxib (GKT137831) was shown to induce remarkable hypoxia-selective cytotoxicity in some HCC cell lines, thus representing a promising drug candidate for cancer therapy [79]. However, the already mentioned study on HCC patients [55] performing histological analyses of NOX isoforms observed that, at variance with NOX2, the expression of NOX4 was lower in mutated hepatocytes with respect to their related non-cancerous ones, whereas increased NOX4 expression could be observed in the nucleus of HCC cells. Although higher levels of both NOX2 and NOX4 in hepatic tumor cells have been positively correlated with liver cirrhosis, further studies are necessary to ascertain whether NOX2 and NOX4 increase is involved in the transition from liver cirrhosis to HCC. It is of extreme interest the observation that shorter patients’ overall survival is correlated with increased expression of NOX4 in cell nuclei and decreased expression in the cytoplasm. This finding may point at NOX4 acting as an oncogene for the promotion of HCC development.

Selected preclinical and clinical studies investigating the role of NOXs in NAFLD, NASH and HCC are reported in Table 1.

## 5. Conclusions

The role of NOXs in the progression and worsening of fatty liver disease, either complicated or not by inflammation, and its transition to liver cancer have been outlined by both preclinical mechanistic studies and clinical observations. Taken together, the results obtained so far indicate that oxidative stress in general and NOX-produced ROS in particular are toxic in non-neoplastic conditions, since they are associated with the establishment of an inflammatory vicious cycle determining and sustaining the transition of NAFLD to NASH. The scenario becomes puzzling when considering neoplastic transition, about which growing evidence indicates that oxidative stress plays both pro- and anti-cancer actions. As far as NOXs are concerned, while the results regarding NOX1 and NOX2 collectively indicate a detrimental effect of their activation, a peculiar role seems to be performed by the NOX4 isoform, whose function in NASH–HCC transition appears controversial, since opposite evidence has been obtained so far. Further studies are warranted to better understand NOX mechanisms and their relationship with cancer signaling pathways and validate their relevance and druggability in neoplastic liver disease prevention and therapy. 

## Figures and Tables

**Table 1:** Preclinical and clinical evidence of the link between modification of NOX expression and activity and oxidative stress in NAFLD, NASH, and HCC.

Study	Model Used	Effect on NOXs	Outcome
**Preclinical studies**			
Yoshida et al. [42]	Rat model of HCC (IP injection of N-diethylnitrosamine (DEN) and high-fat diet (HFD))	The NOX inhibitor Apocynin downregulates p22phox (not p47phox and NOX4) in the hepatocytes of precancerous foci	NOX inhibition suppresses hyperlipidemia and steatosis-induced preneoplastic hepatic lesions
Cremonini et al. [46]	High-fat-diet-fed mice and palmitate-treated HepG2 cells	NOX3/4 increased in palmitate-treated HepG2 cells, as well as redox-sensitive kinases and oxidative stress	(-)-Epicatechin decreases the hepatic expression of NOX3 and NOX4, improving oxidative stress in high-fat-diet fed mice
Bettaieb et al. [47]	High fructose-fed rats	A high-fructose diet upregulates NOX2, p47phox, and NOX4 in the adipose tissue and NOX4 in the liver	(-)-Epicatechin prevents hepatic NOX activation and decreases the upregulation of NOX2 and NOX4, modulating superoxide production in the liver and adipose tissue
Liang et al. [61]	Mouse model of HCC (IP DEN injection in wild-type (WT) C57BL/6 mice and in Nox1^−/−^, Nox4^−/−^, and double knockout mice)	NOX1 expression in macrophages promotes hepatic tumorigenesis through the release of pro-inflammatory cytokines.	Blocking NOX1- and/or NOX1-mediated cytokine release might slow HCC progression. NOX1 ablation in macrophages inhibits cancer growth
Kim et al. [68]	Mouse models of HFD-induced NAFLD: WT C57BL/6 mice, NOX2 (gp91phox)-, TLR4-, MyD88- and Trif-KO mice	NAFLD is reduced in NOX2 (gp91phox)- and TLR4-KO mice	NOX2 deficiency attenuates HFD-induced steatosis and insulin resistance
Sarkar et al. [69]	WT C57BL/6 mice and p47phox KO mice fed with methionine choline-deficient and high-fat diet (MCD-HFD)	Lactate activates NOX2, which mediates cell differentiation and fibrosis	The NOX inhibitor Apocynin inhibits ectopic steatosis
Albadrani et al. [71]	WT C57BL/6 mice and p47phox and miR21 KO mice fed with MCD-HFD	PP2A inhibition exacerbates NAFLD by activating p47phox.	Microcystin-induced NAFLD exacerbation is reduced in p47phox- and miR21-KO mice
Murayama et al. [73]	HCC model in HFD-fed rats	The NOX subunit p22phox is expressed in HFD-induced preneoplastic hepatic foci	α -glycosyl isoquercitrin and spironolactone prevent precancerous lesions, suppress HFD-induced hyperlipidemia and early hepatocarcinogenesis by reducing p67phox and p22phox expression in precancerous lesions
Liu et al. [78]	Healthy immortalized human hepatocytes and HCC cell lines (MHCC-97H, Hep3B, Huh7, MHCC-97L, and HCCLM3)	Hypoxia upregulates NOX4 and prompts ROS-mediated progression and invasion of HCC cells	siRNA-mediated knockdown of NOX4 results in deletion of ROS generation and GLI1-dependent activation and invasion of hypoxic HCC cells
Owada et al. [79]	Hepatic cancer cell lines (HepG2, HLE, and Alexander, PLC/PRF/5)	NOX1/4 inhibition induces selective cytotoxicity and apoptosis of hypoxic cancer cells.	The NOX1/4 inhibitor setanaxib (GKT137831) is able to induce apoptosis in cancer cell, thus representing a promising drug candidate for HCC
Vandierendonck et al. [65]	Hepatic cancer (Huh7, Hep3B Hepa1-6) and monocytic human cell lines, murine macrophages. HCC mouse model (DEN injection)	NOX1 inhibition modulates the polarization of macrophages and affects pro-inflammatory, angiogenic, and fibrotic markers	The NOX1 inhibitor GKT771 reduces inflammation, angiogenesis, and fibrosis
Shiau et al. [72]	Murine HCC model (hepatoma sh-luciferase (Luc) or shHMGB1-ML-14a cells)	NOX2 affects M2 macrophage polarization, sustaining the development of HCC	The HMGB1 and ROS inhibitors ethyl pyruvate and N-acetylcysteine amide decrease M2 macrophage accumulation and liver nodule formation in HCC-bearing mice
Carmona-Cuenca et al. [75]	Primary cultures of rat and human hepatocytes, HepG2, and Hep3B cell lines	TGF-β upregulates NOX4. NOX4 deletion attenuates caspase activation and death of rat hepatocytes	TGF-β displays its proapoptotic activity by the upregulation of NOX4, and its impairment causes HCC cell resistance toward apoptosis
**Clinical studies**			
Rabelo et al. [48]	NAFLD and NASH patients	The rs3017887 SNP of NOX4 is associated with higher ALT concentration in NAFLD patients, the AA genotype in the CYBA-675 T/A CYBA polymorphism with higher triglyceride and lower HDL levels in NASH patients	Genetic NOX polymorphisms are correlated with specific phenotypes in NAFLD/NASH patients
Eun et al. [54]	Liver tissues of HCC patients and healthy subjects	NOX1, NOX2, and NOX5 correlate with metastasis-associated genes, NOX4 and DUOX1 are linked to tumor progression	Higher mRNA levels of NOX4 and DUOX1 correlate with prolonged overall survival, whereas higher levels of NOX1, NOX2, and NOX5 are associated with a poor overall survival
Ha et al. [56]	Liver tissues of HCC patients	In HCC patients, high levels of NOX1 and low levels of NOX4 are observed	NOX1 and NOX4 expression displays an opposite prognostic effect in HCC: high NOX1 and low NOX4 levels correlate with shorter recurrence-free survival and overall survival
Matsumoto et al. [59]	NASH patients	In NASH patients, NOX1 is upregulated in liver sinusoidal endothelial cells (LSECs)	NOX1 upregulation prompts peroxy-nitrite-mediated cellular injury and impairs hepatic microcirculation, helping NAFLD progression
Dou et al. [66]	HCC tissues and cell lines	NOX1 is a downstream target of the tumor suppressor SHMT1 in HCC	NOX1 expression is negatively correlated with SHMT1 expression in HCC
Eun et al. [55]	Liver tissues of HCC patients and healthy subjects	Cytoplasmic NOX2 and nuclear NOX4 expression are increased in HCC cells. NOX4 translocation into the nucleus affects HCC development and progression	NOX2 and NOX4 increased expression in HCC cells correlates with liver cirrhosis. NOX2 and NOX4 could represent diagnostic markers of HCC prognosis
Bettaieb et al. [74]	NAFLD and NASH patients	NASH increases hepatic NOX4 expression, and its deletion in hepatocytes reduces oxidative stress, lipid peroxidation, and liver fibrosis in NASH mice	Hepatic NOX4 deletion is reduces diet-induced hepatic injury and fibrosis. NOX inhibition reduces liver inflammation and fibrosis and increases insulin sensitivity
Chen et al. [57]	HCC patients	High DUOX1 levels in HCC patients correlate with a better prognosis in terms of disease-free survival and overall survival after resection	DUOX1 represents a valuable predictor of HCC patients’ survival
